# Supplementary material for: Circulatory miR-411-5p as a Novel Prognostic Biomarker for Major Adverse Cardiovascular Events in Patients with Atrial Fibrillation
Source: Int J Mol Sci. 2023 Feb 15;24(4):3861. doi: 10.3390/ijms24043861 (PMC9964230; doi:10.3390/ijms24043861)
Supplement: Supplementary file 1 [file ijms-24-03861-s001.zip › ijms-2174012-supplementary.pdf]

Circulatory miR-411-5p as a novel prognostic biomarker for major adverse cardiovascular events in patients with atrial fibrillation

# - Supplementary material

Stephan Nopp[1], M Leontien van der Bent[2], Daniel Kraemmer[1], Oliver Königsbrügge[1], Johann Wojta[3,4], Ingrid Pabinger[1], Cihan Ay[1*], Anne Yaël Nossent[2,3,5*]

* contributed equally as senior authors

[1] Clinical Division of Haematology and Haemostaseology, Department of Medicine I, Medical University of Vienna, Vienna, Austria

[2] Department of Surgery and Einthoven Laboratory for Experimental Vascular Medicine, Leiden University Medical Center, Leiden, the Netherlands

[3] Department of Internal Medicine II, Medical University of Vienna, Vienna, Austria

[4] Ludwig Boltzmann Institute for Cardiovascular Research, Vienna Austria

[5] Department of Laboratory Medicine, Medical University of Vienna, Vienna, Austria

Correspondence: Cihan Ay, MD
Clinical Division of Haematology and Haemostaseology
Department of Medicine I
Medical University of Vienna
Waehringer Guertel 18-20, A-1090 Vienna, Austria
e-mail: cihan.ay@meduniwien.ac.at

SUPPLEMENT

Supplemental Table S1. Outcomes and event-rates in the total study population of patients with atrial fibrillation

	**Count (%)** n= 347	**Rate/100 Person-Years (95% CI)**
**Stroke**	7 (2.0%)	1.6 (0.6-3.2)
**TIA**	2 (0.6%)	0.5 (0.1-2.0)
**Systemic embolism**	2 (0.6%)	0.5 (0.1-2.0)
**Myocardial infarction**	2 (0.6%)	0.5 (0.1-2.0)
**Coronary revascularization without MI diagnosis**	7 (2.0%)	1.6 (0.6-3.2)
**Hospitalization for HF**	5 (1.4%)	1.1 (0.4-2.6)
**Death within one year**	22 (6.3%)	5.0 (3.1-7.5)

Footnote: Confidence intervals were calculated following a Poisson distribution.

Abb.: CI, confidence interval; HF, heart failure; MI, myocardial infarction; TIA, transient ischemic attack

Supplemental Table S2. Results of small RNA sequencing in the discovery case-control study ranked by statistical significance (up to a *p*-value of 0.05)

	log2 FC	log2 CPM	*p*-value	FDR-adj. *p*-value
**hsa-miR-122-3p**	-2.34057672252366	8.87122569844487	0.005659771143828	0.3145010681118
**hsa-miR-375-3p**	-1.32758080224391	9.29190211824105	0.005716590619896	0.3145010681118
**hsa-miR-192-5p**	-1.12992585005836	11.0879189964203	0.008897666162344	0.3145010681118
**hsa-miR-483-3p**	-1.47028489065432	7.62157202871119	0.009549404544209	0.3145010681118
**hsa-miR-1306-5p**	-0.8826729143939	8.81302963580577	0.009855665369124	0.3145010681118
**hsa-miR-99a-5p**	-0.871837634175363	10.2693010320571	0.010255469612341	0.3145010681118
**hsa-miR-140-5p**	1.16020568373112	6.04505572735277	0.024669628906045	0.611230806293342
**hsa-miR-30e-5p**	-1.68223525029586	5.68716665834432	0.028108608655003	0.611230806293342
**hsa-miR-576-5p**	-1.11565326134847	6.39727657105117	0.029897159003479	0.611230806293342
**hsa-miR-21-5p**	0.386597338710552	15.532650290483	0.047319526212891	0.733130082053273
**hsa-miR-432-5p**	1.14275330659164	10.7911040070728	0.047487130960101	0.733130082053273
**hsa-miR-122-5p**	-1.18787289217012	14.8738831598511	0.047812831438257	0.733130082053273

Footnote: The Bioconductor package edgeR was used to investigate differential microRNA expression, using the standard quantile-adjusted conditional maximum likelihood method.

Abb.: FDR, false discovery rate; log2 FC, log base 2 fold change; log2 CPM, log base 2 counts per million

Supplemental Table S3. Results of the subgroup analysis on patients with cardiovascular death and their matched controls in the discovery study ranked by statistical significance (up to a *p*-value of 0.05)

	log2 FC	log2 CPM	*p*-value	FDR-adj. *p*-value
hsa-miR-150-5p	-2.019024579	12.80098332	6.71E-05	0.012472796
hsa-miR-127-3p	1.794688616	13.34400397	0.000233064	0.019430654
hsa-miR-1908-5p	2.222607287	7.9720971	0.000313398	0.019430654
hsa-miR-451a	-1.303909995	18.08653029	0.003033158	0.141041861
hsa-miR-4446-3p	1.475300796	10.28791453	0.004608483	0.144173243
hsa-miR-15a-5p	-1.646259024	8.7761774	0.00465075	0.144173243
hsa-miR-744-5p	1.432595549	9.404320034	0.006377248	0.169452587
hsa-miR-625-5p	1.47081314	8.771415038	0.008792127	0.182913819
hsa-miR-142-5p	-1.854638694	7.635067182	0.009710023	0.182913819
hsa-miR-483-3p	-1.981287021	7.704546454	0.009834076	0.182913819
hsa-miR-10b-5p	-1.241384877	9.032492953	0.011020184	0.186341286
hsa-miR-139-3p	1.157749549	10.97401451	0.016312053	0.252836824
hsa-miR-411-5p	1.110253412	10.08640168	0.020237193	0.269499902
hsa-miR-485-5p	1.121649128	10.61942484	0.020284939	0.269499902
hsa-miR-432-5p	1.769794467	11.21822575	0.030483713	0.31818657
hsa-miR-409-3p	1.036251704	9.671392053	0.032557606	0.31818657
hsa-miR-144-3p	-2.335195896	6.421698524	0.036869414	0.31818657
hsa-miR-23b-3p	0.848353167	11.24695279	0.037131829	0.31818657
hsa-miR-151a-5p	0.82745551	12.8139468	0.037979281	0.31818657
hsa-miR-337-3p	1.016599165	8.845465787	0.039146621	0.31818657
hsa-miR-16-5p	-0.74406623	12.63382169	0.039239845	0.31818657
hsa-miR-194-5p	-1.181265324	8.535488459	0.039700666	0.31818657
hsa-miR-375-3p	-1.070980578	9.527332313	0.042139871	0.31818657
hsa-miR-15b-5p	-0.969119875	10.79390341	0.042437827	0.31818657
hsa-miR-5100	1.115298684	7.611960921	0.043356951	0.31818657
hsa-miR-200a-3p	-1.945443645	5.362914519	0.044477693	0.31818657

Footnote: The Bioconductor package edgeR was used to investigate differential microRNA expression, using the standard quantile-adjusted conditional maximum likelihood method.

Abb.: FDR, false discovery rate; log2 FC, log base 2 fold change; log2 CPM, log base 2 counts per million

Supplemental Table S4. Assays used for reverse-transcription quantitative polymerase chain reaction (RT-qPCR)

Assays	Company	Assay ID & Catalog number
miRNeasy Serum/Plasma Kit	Qiagen	Catalog number: 217184
TaqMan™ MicroRNA Reverse Transcription Kit	ThermoFisher	Catalog number: 4366596
TaqMan™ Universal PCR Master Mix	ThermoFisher	Catalog number: 4304437
TaqMan™ MicroRNA Assay hsa-miR-483-3p	ThermoFisher	Assay ID 002339<br>Catalog number: 4427975
TaqMan™ MicroRNA Assay hsa-miR-150-5p	ThermoFisher	Assay ID 000473<br>Catalog number: 4427975
TaqMan™ MicroRNA Assay hsa-miR-127-3p	ThermoFisher	Assay ID 000452<br>Catalog number: 4427975
TaqMan™ MicroRNA Assay hsa-miR-1908-5p	ThermoFisher	Assay ID 121109_mat<br>Catalog number: 4427975
TaqMan™ MicroRNA Assay hsa-miR-15a-5p	ThermoFisher	Assay ID 000389<br>Catalog number: 4427975
TaqMan™ MicroRNA Assay hsa-miR-411-5p	ThermoFisher	Assay ID 001610<br>Catalog number: 4427975
TaqMan™ MicroRNA Assay hsa-miR-625-5p	ThermoFisher	Assay ID 002431<br>Catalog number: 4427975
TaqMan™ MicroRNA Assay cel-miR-39-3p	ThermoFisher	Assay ID 000200<br>Catalog number: 4427975
